# The self-regulation of neurotransmitter release

**DOI:** 10.3389/fncel.2014.00181

**Published:** 2014-06-27

**Authors:** Marco Canepari

**Affiliations:** ^1^Inserm U836, Grenoble Institute of NeuroscienceGrenoble, France; ^2^Team MOTIV, Laboratoire Interdisciplinare de Physique (CNRS UMR 5588), Université Joseph FourierGrenoble, France; ^3^Laboratories of Excellence, Ion Channel Science and Therapeutics

**Keywords:** leech, serotonin, Retzius, calcium signaling, neurotransmitter release

Retzius cells are large neurons in the ganglia of the leech (Lent, 1977), releasing 5-HT (Leake, 1986) and orchestrating the animal behavior (Carretta, 1988). The leech nervous system is relatively simple and its exploration allows correlating, in a quantitative manner, the chemical and electrical activity, at cellular level, with the animal behavior (Mazzoni et al., 2007).

The recent work of Francisco De-Miguel, Citlali Trueta and colleagues has given important contributions on the understanding of 5-HT secretion at Retzius cells that occurs both at the site of synaptic targets and from the soma (De-Miguel and Trueta, 2005). The somatic 5-HT release is dependent on L-type calcium channel activation (Trueta et al., 2003) and on calcium-induced calcium release (Trueta et al., 2004). Retzius cells have 5-HT autoreceptors and the activation of these proteins is coupled to chloride channels producing synaptic autoinhibition (Cercós et al., 2009).

In the article by Leon-Pinzon et al. (2014) recently published in *Frontiers in Cellular Neuroscience*, a significant progress on the understanding of the mechanisms induced by 5-HT autoreceptors is presented. Specifically, the authors report the very interesting finding that sustained 5-HT vesicular release is regulated by a positive feedback at the same Retzius cell. The soma of Retzius neurons releases serotonin from dense clusters of core vesicles without evidence of active zones. This type of secretion is similar to secretion by excitable endocrine cells. When 5-HT release is associated with high-frequency action potential firing, the initial 5-HT release mediated by calcium entry via voltage-gated calcium channels activates 5-HT autoreceptors. This activation leads to one or two additional episodes of intracellular calcium increase which amplify the release of 5-HT. In order to perform this study, the authors used an elegant experimental analysis combining electrophysiological recordings, fluorescence imaging of calcium and neurotransmitter activity and pharmacological tests.

The physiological amplification mechanism reported in this paper allows Retzius cells operating in a two-state mode: a “weakly releasing” mode and a “strongly releasing” mode. Presumably, there will be a critical frequency that may vary from cell to cell switching from one mode to the other. This bi-stable behavior, which might be similar to what reported in another invertebrate neuron (Achenbach et al., 1997), plays an obviously important role in the computational properties of both Retzius cells and associated neuronal networks. But did we learn something that can be possibly extended to the mammalian nervous system? The leech nervous system represents a rich model for vertebrate systems from the points of view of both cellular neurobiology (Schmold and Syed, 2012) and of higher functions (Sahley, 1995). In serotonergic neurons in the raphe nuclei of the rodent brain, 5-HT is also released from the soma (Sarkar et al., 2012) and from the dendrites (Colgan et al., 2012). These phenomena have several common features with 5-HT somatic release at Retzius neurons. Thus, Leon-Pinzon et al. (2014) formulate the hypothesis that the mechanisms of self-regulation of 5-HT discovered in Retzius neurons may also operate in the mammalian nervous system, perhaps by activating receptors not only at the soma and at different time-scale.

## Conflict of interest statement

The author declares that the research was conducted in the absence of any commercial or financial relationships that could be construed as a potential conflict of interest.

## References

[B1] Achenbach H. Walther C. Wicher D. (1997). Octopamine modulates ionic currents and spiking in dorsal unpaired median (DUM) neurons. Neuroreport 8, 3737–3741 10.1097/00001756-199712010-00016 9427361

[B2] Carretta M. (1988). The Retzius cells in the leech: a review of their properties and synaptic connections. Comp. Biochem. Physiol. A Comp. Physiol. 91, 405–413 10.1016/0300-9629(88)90611-1 2906825

[B3] Cercós M. G. De-Miguel F. F. Trueta C. (2009). Real-time measurements of synaptic autoinhibition produced by serotonin release in cultured leech neurons. J. Neurophysiol. 102, 1075–1085 10.1152/jn.00107.2009 19535486

[B4] Colgan L. A. Cavolo S. L. Commons K. G. Levitan E. S. (2012). Action potential-independent and pharmacologically unique vesicular serotonin release from dendrites. J. Neurosci. 32, 15737–15746 10.1523/JNEUROSCI.0020-12.2012 23136413 PMC3505755

[B5] De-Miguel F. F. Trueta C. (2005). Synaptic and extrasynaptic secretion of serotonin. Cell. Mol. Neurobiol. 25, 297–312 10.1007/s10571-005-3061-z 16047543 PMC11529639

[B6] Leake L. D. (1986). Leech Retzius cells and 5-hydroxytryptamine. Comp. Biochem. Physiol. C. 83, 229–239 10.1016/0742-8413(86)90116-7 2871982

[B7] Lent C. M. (1977). The Retzius cells within the central nervous system of leeches. Prog. Neurobiol. 8, 81–117 10.1016/0301-0082(77)90012-0 320629

[B7a] Leon-Pinzon C. Cercós M. G. Noguez P. Trueta C. De-Miguel F. F. (2014). Exocytosis of serotonin from the neuronal soma is sustained by a serotonin and calcium-dependent feedback loop. Front. Cell. Neurosci. 8:169 10.3389/fncel.2014.00169 PMC4072984 25018697

[B8] Mazzoni A. Broccard F. D. Garcia-Perez E. Bonifazi P. Ruaro M. E. Torre V. (2007). On the dynamics of the spontaneous activity in neuronal networks. PLoS ONE 2:e439 10.1371/journal.pone.0000439 17502919 PMC1857824

[B9] Sahley C. L. (1995). What we have learned from the study of learning in the leech. J. Neurobiol. 27, 434–445 10.1002/neu.480270314 7673899

[B10] Sarkar B. Das A. K. Arumugam S. Kaushalya S. K. Bandyopadhyay A. Balaji J. (2012). The dynamics of somatic exocytosis in monoaminergic neurons. Front. Physiol. 3:414 10.3389/fphys.2012.00414 23133421 PMC3490137

[B11] Schmold N. Syed N. I. (2012). Molluscan neurons in culture: shedding light on synapse formation and plasticity. J. Mol. Histol. 43, 383–399 10.1007/s10735-012-9398-y 22538479

[B12] Trueta C. Méndez B. De-Miguel F. F. (2003). Somatic exocytosis of serotonin mediated by L-type calcium channels in cultured leech neurones. J. Physiol. 547, 405–416 10.1113/jphysiol.2002.030684 12562971 PMC2342656

[B13] Trueta C. Sánchez-Armass S. Morales M. A. De-Miguel F. F. (2004). Calcium-induced calcium release contributes to somatic secretion of serotonin in leech Retzius neurons. J. Neurobiol. 61, 309–316 10.1002/neu.20055 15389693

